# Immunological Analysis of People in Northeast China after SARS-CoV-2 Inactivated Vaccine Injection

**DOI:** 10.3390/vaccines9091028

**Published:** 2021-09-16

**Authors:** Yu Fu, Fang Chen, Lifen Cui, Yue Zhao, Henan Zhang, Shuang Fu, Jihong Zhang

**Affiliations:** Hematology Laboratory, Shengjing Hospital of China Medical University, Shenyang 110022, China; fuyu_727@126.com (Y.F.); chenfang020117@163.com (F.C.); cuilf@sj-hospital.org (L.C.); zy_3026@126.com (Y.Z.); cmunancy9226428@126.com (H.Z.); coolerfspretty@126.com (S.F.)

**Keywords:** SARS-CoV-2, inactivated vaccines, lymphocyte subsets, cytokines, neutralizing antibodies

## Abstract

Clarifying changes in the immune microenvironment caused by vaccination is crucial for the development and application of vaccines. In this study, we analyzed seroconversion of antibodies, 12 key cytokines, and 34 lymphocyte subsets at three time points (D-1, D14, and D42) around vaccination and differences between two inactivated vaccines (Sinopharm and Sinovas) to understand the immune response induced by inactivated vaccines in the real world. The results showed that 62.5% and 75% of the participants achieved neutralizing antibody seroconversion on D14 and D42, respectively. After vaccination, IL-5 and IL-6 increased, and INF-γ decreased. IL6, IL-1B, INF-γ, IL-8, and IL-12p70 showed statistical significance in the comparison of different groups. In terms of lymphocyte subsets, CD3 +, CD56 +, CD3 + CD8 +, CD8 + CD71 +, and CD56 + CD71 + showed upward trend, while CD19 +, CD4 + CD8 +, CD8 + CD45RA +, CD4 + HLA-DR +, CD8 + HLA-DR +, and CD8 + CD38 + showed downward trend. Additionally, we found certain differences between the two vaccines in neutralizing antibodies, cytokines, and lymphocyte subsets. This research is a clinical observation on the immune response after vaccination through detecting various immune indicators, which showed that the inactivated vaccines induced both humoral immunity by producing neutralizing antibodies and cellular immunity. The cellular immunity induced by these two vaccines was a Th2-biased response, and it may also lead to a mild Th1-type response.

## 1. Introduction

Since the outbreak of COVID-19 (coronavirus disease 2019) pandemic caused by severe acute respiratory syndrome coronavirus 2 (SARS-CoV-2) in December 2019, the impact of the epidemic on human activities has been aggravating. As of early July 2021, over 183 million people worldwide had been confirmed infected with SARS-CoV-2, of which 3.9 million had died [1]. Although the epidemic had been effectively controlled in the Chinese mainland, there were signs of slight rebounds mainly caused by the spread of imported cases or products and food that were exposed to the SARS-CoV-2 virus. The well-acknowledged solution to such a pandemic is global herd immunity based on vaccination. Currently, a total of 102 vaccine projects worldwide are ongoing, including inactivated vaccines, adenovirus-vectored vaccines, mRNA vaccines, recombinant protein vaccines, etc. There are 26 vaccines in phase 3 or phase 2/3 clinical trials and five in phase 4 clinical trials. In terms of inactivated vaccines, eleven projects have entered clinical trials, of which seven are in phase 1or 2 clinical trials, eight are in phase 3 or 2/3, and one is in phase 4 [2]. The two inactivated vaccines involved in this study are CoronaVac from Sinovac Research and Development Co. Ltd. (hereinafter referred to as Sinovas vaccine) and BBIBP-CorV from Sinopharm, China National Biotec Group Co and Beijing Institute of Biological Products (hereinafter referred to as Sinopharm vaccine), which were the most widely used and earliest in clinical trials in China. These two vaccines received the WHO emergency use grant [3].

Currently, there are few studies on the changes in the immune microenvironment caused by SARS-CoV-2 vaccines. The few existing ones focus on mRNA or adenovirus-vector vaccines. Most of these vaccines induce a Th1-biased cellular immunity response. Studies on inactivated vaccination are rare. Phase 3 trials of Sinovas vaccine in Chile and Turkey suggest that the inactivated SARS-CoV-2 vaccines effectively prevent COVID-19 along with a good safety profile, but the magnitudes of T-cell responses are not mentioned [4,5]. This might be because inactivated vaccines only induce humoral immunity but not cellular immunity. However, it is controversial in actual vaccine development and application. With suitable adjuvants, partially inactivated vaccines could also induce cellular immunity. Balanced cellular and humoral immune responses are important to defend against the virus [6]. Therefore, it is particularly significant to clarify the immune changes induced by an inactivated vaccine to evaluate the effect of vaccines.

Vaccinations programs in China have made great progress since March 2021, and comparative evaluations are important to push the programs forward. We analyzed the seroconversion changes of antibodies, cytokines, and lymphocyte subsets in participants before and after vaccination of the two vaccines to explore the immunity characteristics induced by the vaccines in the northeast China population.

## 2. Materials and Methods

### 2.1. Participants and Study Design

The 33 participants were medical staff from Shengjing Hospital of China Medical University; they were tested at least three times for SARS-CoV-2 nucleotide by real-time PCR to ensure none were infected with the virus before the study. Physical conditions and basic information were recorded continuously since vaccination to evaluate the safety of the vaccines. The participants were randomly vaccinated with the Sinopharm vaccine or Sinovas vaccine. Both vaccines were injected in a two-dose schedule on day 0 and day 21 as 0.4 μg/0.5 ml per injection for the Sinopharm vaccine and 0.3 μg/0.5 ml for the Sinovas vaccine. Peripheral blood samples were sampled in EDTA anticoagulant tubes and serum separator tubes on the day before the first injection (D-1), 14 days after the first injection (D14), and 21 days after the second injection (D42). The collection time points were determined according to the best antibody acquisition time of phase 1/2 clinical data of Sinopharm and Sinovas vaccines [7,8]. All samples were processed within 4 h after sampling. Serum separator tubes were processed for the collection of blood serum. EDTA anticoagulant tubes were processed for the collection of blood plasma. Written informed consent was obtained from all participants. The study was approved by the Ethics Committee of Shengjing Hospital of China Medical University—agreement NO. 2021PS454K, and all participants gave written informed consent before the studies were performed.

### 2.2. SARS-CoV-2 S1 RBD IgG, IgM Antibody Specific ELISA

Blood samples were taken on D-1, D14, and D42 to measure the serum SARS-CoV-2 S1 receptor-binding domain (RBD)-specific IgG/IgM antibodies using the ELISA method to explore the basic antibody level and antibody acquisition status. ELISA kit was from Cusabio Biotechnology China. The microtiter plate was pre-coated with human SARS-CoV-2 S1 RBD. Diluted serum (1:100) was pipetted into the wells and incubated for 30 min at 37 °C. Each well was aspirated and washed; the process was repeated two times for a total of three washes. Then, 100 μL of HRP-conjugate (anti-human IgG or IgM conjugated) was added into each well and incubated for 30 min at 37 °C, and 90 μL TMB substrate was added after a further washing step five times. Development was performed using stop solution, and optical density was measured at 450 nm. Seroconversion was defined as OD_sample_ ≥ 2.1 × OD_negative_.

### 2.3. SARS-CoV-2 Neutralizing Antibody ELISA

Blood samples were taken on D-1, D14, and D42 to measure the serum SARS-CoV-2 neutralizing antibody using the ELISA method to determine the basic antibody level and antibody acquisition status. ELISA kits were from Cusabio Biotechnology China. The microtiter plate was pre-coated with SARS-CoV-2 RBD. After pipetting diluted serum (1:100) from participants into the wells for a 37 °C incubation, horseradish peroxidase (HRP)=conjugated ACE2 was added to the appropriate microtiter plate wells. Then, substrate solution was added to the wells, and the color developed inversely to the quantity of SARS-CoV-2 neutralizing antibody in the sample. The color development was stopped using a stop solution, and the intensity of the color was measured at 450 nm. Seroconversion was defined as (1-(OD_sample_-OD_blank_))/OD_average value of participants before vaccination_ ≥ 20%.

### 2.4. Lymphocyte Subsets

Immunophenotype of lymphocyte subsets was performed with flow cytometry (DxFLEX; Beckman Coulter, Suzhou, China) using mouse anti-human fluorescent monoclonal antibodies (fluorescein isothiocyanate [FITC], phycoerythrin [PE], R-Phycoerythrin-Texas Red^®^-X [ECD], phycocerythrin cyanin 5 [PC5], R Phycoerythrin-Cyanine 5.5 [PC5.5], Phycoerythrincyanin 7 [PC7], and allophycocyanin [APC], APC-Alexa fluor 700 [APC-AF700], APC-Alexa fluor 750 [APC-AF750]). The antibodies were purchased from Beckman Coulter (Miami, FL, USA) and BD Pharmingen (San Diego, CA, USA). Fresh peripheral blood (2 ml) was anticoagulated with EDTA and labeled with fluorescein, as shown below: CD57-FITC/CD56-PE/CD4-ECD/CD8-PC5.5/CD3-PC7/ HLA-DR -APC/CD38-APC-AF700/CD45-APC-AF750; CD45RO-FITC/CD45RA-PE/CD4-ECD/ CD8-PC5; 5/CD3-PC7/CD71-APC/CD56-APC-AF700/CD45-APC-AF750; CD20-FITC/ CD27-PE/CD5 -PC5.5/CD19-PC7/CD38-APCA700/CD45-APCA750; CD28-FITC/CD8-PE/ CD3-PC5; CD29-FITC/CD4-PE/CD3-PC5; CD16-FITC/CD56-PE/CD3-PC5; CD38-FITC/CD24-PE/ CD19-PC5; CD4-FITC/CD127-PE/CD25-PC5. After incubation in the dark for 15 min, hemolysis was achieved with hemolysin (Beckman OptiLyse C lysing solution) for 10 min. At least 40,000 lymphocytes cells were collected, and the data were analyzed with Kaluza (Beckman Coulter). The lymphocyte subsets for statistics included CD3+, CD3+CD4+, CD3+CD8+, CD56+, CD19+, CD4+ CD38+, CD8+CD38+, CD4+CD57+, CD8+CD57+, CD56+CD57+, CD4+CD8+, CD4+HLA-DR+, CD8+ HLA-DR+, CD19+CD71+, CD3+CD71+, CD4+CD71+, CD8+CD71+, CD56+CD71+, CD4+CD45RO+, CD4+CD45RA+, CD8+CD45RO+, CD8+CD45RA+, CD19+ CD27+, CD19+ CD27−, CD19+/dim CD27+CD38++, CD3+CD8+CD28+, CD3+CD8+CD28−, CD4+CD25+ CD127dim, CD38+CD24+CD19+, CD3+CD4+CD29+, CD3+CD4+CD29−, CD3+ CD56+ NKT, CD56+CD16+, and CD56+CD16−.

### 2.5. Cytokines

The analysis of cytokines was performed by flow cytometry using the microsphere capture method (Raise care, Qingdao, China). The plasma samples mixed with buffer and capture microsphere antibodies were incubated for 2 h with shaking in the dark, then SA-PE was added and incubated for 30 min with shaking in the dark. After washing and resuspending, a flow cytometer was used to test the samples. A total of 12 cytokines were detected, including IL-1β, IL-2, IL-4, IL-5, IL-6, IL-8, IL-10, IL-12p70, IL-17, IFN-γ, IFN- α, and TNF-α. At least 1100 microspheres for each sample were collected to ensure the accuracy of the data, and LEGENDplex8.0 analysis software was used for data analysis.

### 2.6. Statistic

The data was displayed as mean ± SD or Median, all statistical tests were two-sided, and the significance level was set at *p* values of 0.05 or less for inferential analysis. For normally distributed data, differences between groups at a specific time point were tested with unpaired *t*-test or non-parametric Mann–Whitney test. The measurement data conforming to the normal distribution and uniform variance were tested by *t*-test, and the non-parametric Mann–Whitney test was used for non-conforming measurement data. Statistical analysis was performed using GraphPad Prism 8 (GraphPad Software) and SPSS 21.0 (IBM). There were 33 participants in this study; due to the withdrawal of some participants, 32 participants actually participated in the detection of specific antibodies, 31 in the detection of cytokines, and 33 in the detection of lymphocyte subsets.

## 3. Results

### 3.1. Baseline Characteristics of Participants

Healthy adults aged 28–47 (*n*  =  33) were randomized to receive either Sinopharm or Sinovas vaccines. Blood samples were collected on D-1, D14, and D42. The age and gender distribution of the participants are shown in Table 1. Since the vaccination was only available for participants aged 18–59 at the time we started the research, all participants in this study were within this age range. For the small sample size in this study, the participants were mostly female; the correlation analysis of age and gender of vaccine effect was not conducted.

### 3.2. RBD IgG, IgM, and Neutralizing Antibody Expression Induced by Inactivated Vaccine

**Figure 1:**
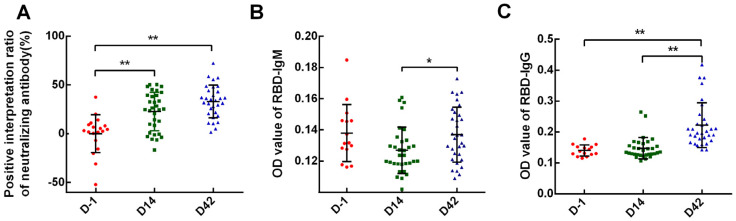
SARS-CoV-2 specific antibodies of each time-point. Neutralizing antibodies, RBD IgM, and IgG antibodies were examined on D-1, D14, and D42 after vaccination. (**A**) neutralizing antibodies; (**B**) RBD IgM antibodies; (**C**) RBD IgG antibodies. Data points show mean ± SD; the error bars reflect SD. * *p* < 0.05; ** *p* < 0.01.

To explore the positive rate of neutralizing antibodies and the continuity of antibody acquisition of the two inactivated vaccines, we applied ELISA to detect specific neutralizing antibodies levels on 32 participants. According to the interpretation criteria of the test kit, the positive rate of the inactivated vaccines was 62.5% (20/32) on D14 and 75% (24/32) on D42. At the observational endpoint (D42) of this study, 58.3% (7/12) participants with Sinopharm vaccine and 85.0% (17/20) participants with Sinovas vaccine got neutralizing antibody seroconversion. Among the results, the antibody levels on D14 (*p* = 0.001) and D42 (*p* = 0.000) were higher than D-1 and showed a rising trend, but not all participants showed an upward trend of antibody levels after receiving the second dose. In this study, we found that antibody levels in 62.5% (20/32) participants were increased on D42 comparing with D14, but the remaining 37.5% (12/32) showed a downward trend. In each group, there was one participant who did not reach antibody-positive levels either on D14 or D42, but the difference was not statistically significant (*p* = 1.000) (Figure 1A).

While testing the neutralizing antibodies, we also detected RBD-specific IgG and IgM antibodies at the same time, which showed no positive IgM antibody in all participants at the three time points. As for IgG antibodies, the total positive rate after receiving the second dose was 15.62% (5/32), for Sinopharm vaccines was 8.33% (1/12) and for Sinovas vaccines 20.00% (4/20); the difference was not statistically significant (*p* = 0.6399). In addition, although the IgG antibody level did not reach a positive cut-off value, when setting aside the judgment of positivity interpretation, the antibody level of IgG on D42 showed a significant increase compared with D-1 (*p* = 0.000) and D14 (*p* = 0.000), indicating that there was still an antibody response after the second dose (Figure 1B,C).

### 3.3. T-cell Responses Were Biased to a Th2 Phenotype after Inactivated Vaccine Injection

**Figure 2:**
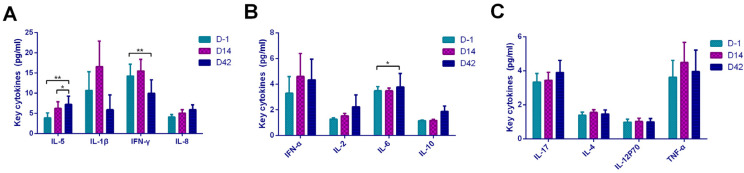
Key cytokines of each time point. Key cytokines, containing IL-5, IFN-α, IL-2, IL-6, IL-1β, IL-10, IFN-γ, IL-8, IL-17, IL-4, IL-12P70, and TNF-α were examined on D-1, D14, and D42 after vaccination. (**A**) IL-5, IL-1β, IFN-γ, and IL-8; (**B**) IFN-α, IL-2, IL-6, and IL-10; (**C**) IL-17, IL-4, IL-12P70, and TNF-α. Data points show mean ± SD; the error bars reflect SD. * *p* < 0.05; ** *p* < 0.01.

To explore whether the vaccination caused cellular responses and the type of the response, we performed flow cytometry analysis of 12 cytokines including IL-5, IFN-α, IL-2, IL-6, IL-1β, IL- 10, IFN-γ, IL-8, IL-17, IL-4, IL-12P70, and TNF-α (Figure 2). The results revealed that IL-5 (*p* = 0.027, *p* = 0.008) (Figure 2A) and IL-6 (*p* = 0.023) (Figure 2B) were increased, while IFN-γ (*p* = 0.01) (Figure 2A) was decreased after vaccination. To avoid the illusion of the appeal trend caused by the difference between the two vaccines, we compared the cytokine between two vaccines groups on D-1, D14, and D42 and observed no statistically differences except IL-1β (*p* = 0.023), which was higher in the Sinopharm vaccine on D42.

Based on the above results, we concluded that vaccination activated the Th2 immune cells through increased IL-5 and IL-6. Although IFN-γ was differentially expressed, it showed a downward trend after vaccination, and it would not activate the Th1-dominated cellular immune response.

### 3.4. Comparison of Cytokines in Different Groups of Neutralizing Antibody Level

**Figure 3:**
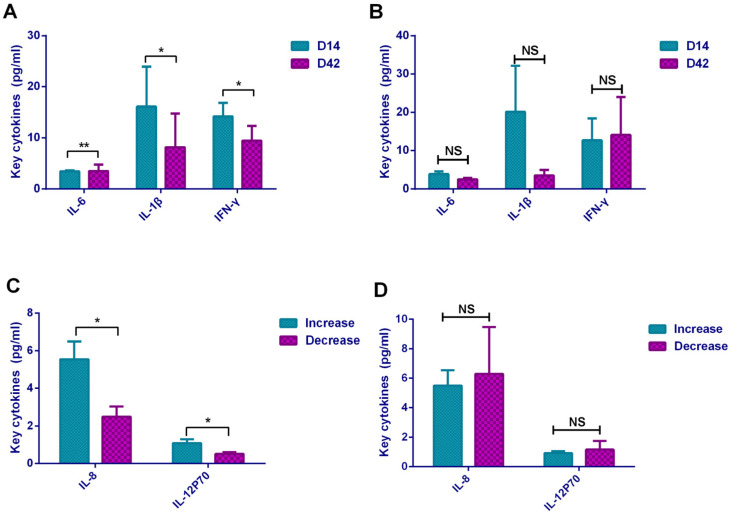
Comparison of key cytokines in different groups. Key cytokines contained IL-5, IFN-α, IL-2, IL-6, IL-1β, IL-10, IFN-γ, IL-8, IL-17, IL-4, IL-12P70, and TNF-α. (**A)** In increased group, comparing the differences of cytokines between the two time points D14 and D42. (**B**) In decreased group, comparing the differences of the cytokines between the two time points D14 and D42. (**C**) Comparing the differences of each cytokine between the increased group and the decreased group on D14. (**D**) Differences of each cytokine between the increased group and the decreased group on D42. * *p* < 0.05; ** *p* < 0.01; NS—no statistically significant differences.

The neutralizing antibody levels on D14 and D42 after vaccination appeared in two different trends. Therefore, we grouped all the subjects by neutralizing antibody levels into the increased group (*n* = 20) and decreased group (*n* = 12), the cytokine differences between the two groups were analyzed. The results showed that the level of each cytokine between D14 and D42 in the decreased group were not different (Figure 3B); while IL-6 (*p* = 0.004) showed an upward trend, IL-1β (*p* = 0.033) and IFN-γ (*p* = 0.020) showed a downward trend in the increased group (Figure 3A). In addition, from another angle, we analyzed the differences of 12 cytokines between the increased group and the decreased group on D14 and D42, respectively, which revealed that on D14, the expression levels of IL-8 (*p* = 0.049) and IL-12P70 (*p* = 0.020) in the increased group were significantly higher than those in the decreased group (Figure 3C), while on D42 for each cytokine there was no statistically significant difference between the two groups (Figure 3D).

### 3.5. Changes of Lymphocyte Subsets after Vaccination

**Figure 4:**
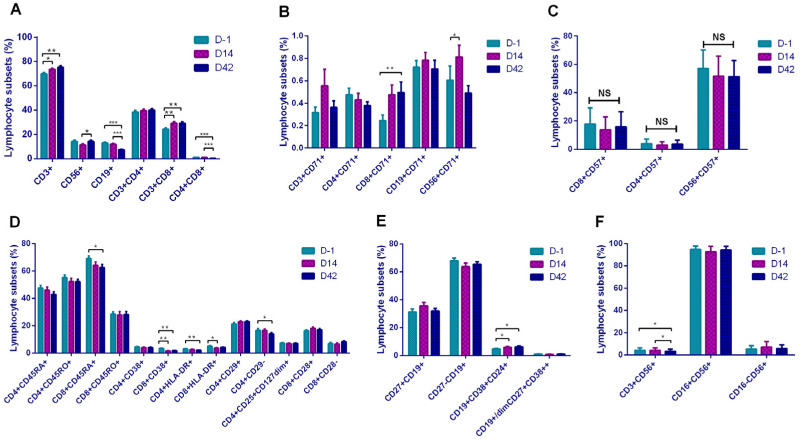
Comparison of lymphocyte subsets between 3 time points: (**A**) basic lymphocyte subsets; (**B**) activated lymphocyte subset (CD71+); (**C**) proliferating lymphocyte subsets (CD57+); (**D**) T-lymphocytes subsets; (**E**) B-lymphocyte subsets; (**F**) NK cell subsets. * *p* < 0.05; ** *p* < 0.01; *** *p* < 0.001; NS—no statistically significant differences.

To further evaluate the changes in organism immune status caused by vaccination, we used flow cytometry to analyze the 33 participants’ lymphocyte subsets including the basic grouping of lymphocytes, activated lymphocytes, proliferating lymphocytes, T lymphocytes, B lymphocytes, and NK cells on D-1, D14, and D42 (Figure 4).

We found that among the basic lymphocyte subsets, NK cells, B lymphocyte, suppressor T cell (Ts), and double-positive T cell (CD4+CD8+, DPT) subsets underwent statistically significant changes after vaccination (Figure 4A). Among them, NK cells showed an increasing trend on D42 compared with D14, B lymphocytes showed a decreasing trend on D42 compared with D-1 and D14, Ts cells were increased on D14 and D42 compared with D-1, while DPT cells showed a decreasing trend both on D14 and D42 compared to D-1. CD71+Ts cells were significantly increased on D42 (Figure 4B). There were no statistically significant differences in CD57+ cell subsets (Figure 4C). In the grouping of T-lymphocytes, naive T cells decreased on D42, and CD8+CD38+, CD4+HLA-DR+, and CD8+HLA-DR+ subsets also showed a downward trend after vaccination (Figure 4D). In B lymphocyte subsets, Breg cells (CD19+CD38+CD24+) showed higher levels on D14 and D42 than D-1 (Figure 4E). Among the NK cell subsets, NKT cells showed a downward trend on D42 compared to D-1 and D14 (Figure 4F).

### 3.6. Comparison of Lymphocyte Subsets between Sinopharm and Sinovas Inactivated Vaccines

**Figure 5:**
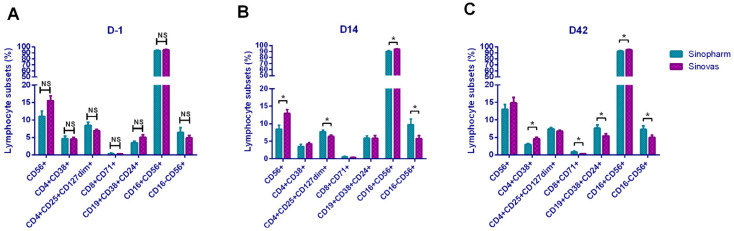
Comparison of lymphocyte subsets between two vaccines: (**A**) basic lymphocyte subsets on D-1; (**B**) lymphocyte subsets on D14; (**C**) lymphocyte subsets on D42. * *p* < 0.05; ** *p* < 0.01; NS—no statistically significant differences.

The participants were randomly vaccinated with two inactivated vaccines produced in China. Here, we compared the alterations of lymphocyte subsets induced by these two vaccines. By comparing the 34 lymphocyte subsets between the two groups on D-1, we determined baseline values of the lymphocyte subsets, which showed no differences at initial status (Figure 5A). On D14, statistical differences in NK cells, Treg cells, CD16+NK cells, and CD16-NK cells were noted (Figure 5B), and on D42, we observed statistical differences of CD4+CD38+, CD71+Ts cells, Breg cells, CD16+NK cells, and CD16-NK cells between the two groups (Figure 5C). The Sinopharm vaccine induced higher Treg cells level on D14, higher Ts cells, and Breg cells level on D42, while the Sinovas vaccine induced higher activated NK cells level on D14, higher activated Th, and activated NK cells on D42.

### 3.7. Comparison of Lymphocyte Subsets in Different Groups of Neutralizing Antibody Level

**Figure 6:**
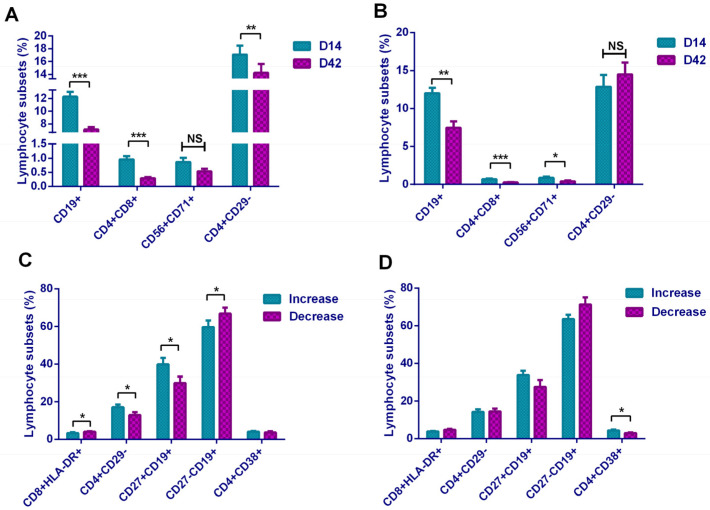
Comparison of lymphocyte subsets in different groups: (**A**) in increased group, comparing the differences of lymphocyte subsets between the two time points D14 and D42; (**B**) in decreased group, comparing the differences of lymphocyte subsets between the two time points D14 and D42; (**C**) comparing the differences of lymphocyte subsets between the increased group and the decreased group on D14; (**D**) comparing the differences of lymphocyte subsets between the increased group and the decreased group on D42. * *p* < 0.05; ** *p* < 0.01; *** *p* < 0.001; NS—no statistically significant differences.

We also analyzed lymphocyte subsets of the two groups. In the increased group, B lymphocyte, DPT cells, and CD4+CD29− subsets showed a downward trend on D42 compared with D14 (Figure 6A), while in the decreased group, the B lymphocytes, DPT cells, and CD56+CD71+ subsets also showed a downward trend (Figure 6B). Since B lymphocyte and DPT cells showed the similar trend in the overall comparison, here we concluded that the differences of these two lymphocyte subsets and the neutralizing antibody levels were not relevant. Therefore, in these comparisons, it was believed that the level of CD4+CD29− subset was negatively correlated with the level of neutralizing antibody, and the level of CD56+CD71+ subset was positively correlated with the level of neutralizing antibody.

In addition, comparing from another angle, we analyzed the differences of 34 lymphocyte subsets between the increased group and the decreased group on D14 and D42, respectively. It was found that on D14, CD8+HLA-DR+ and CD27−CD19+ subsets had a higher proportion in the decreased group, while CD4+CD29− and CD27+CD19+ subsets had a higher proportion in the increased group (Figure 6C). On D42, only the CD4+CD38+ subset was different between the two groups, which manifested as a higher proportion of this subset in the antibody-increased group (Figure 6D). It was suggested that CD4+CD29−, CD27+CD19+, and CD4+CD38+ subsets promoted the maintenance of neutralizing antibody levels, while CD8+HLA-DR+ and CD27−CD19+ subsets may perform the opposite effect.

## 4. Discussion

In our study, 62.5% and 75% of the overall participants achieved neutralizing antibody seroconversion on D14 and the observational endpoint (D42), respectively, which was 83.3%(D14)/58.3%(D42) of Sinopharm and 50%(D14)/85%(D42) of Sinovas Vaccines. This was lower than 75%(D14)/100%(D28) and 92.4%(D14)/94.1–97.4%(D28) that reported in phase 2 clinical trials [7,8] (Table S1). The results indicated that both inactivated vaccines had moderate immunogenicity. However, it is worth noting that IgM antibodies were not tested at any time point by the ELISA method, and the overall positive rate of IgG was only 15.62%. In the Phase 1/2 clinical trials of the two vaccines, none of the IgM positive rates were involved. For IgG, the Sinovas vaccine reported their positive rates at 96.5–99.2% [7] and Sinopharm at 100% [9]. We analyzed the potential reasons for the differences: (1) different detection methods and positive standards; (2) different detecting time points. The peak of IgM and IgG generally occurs respectively on days 7–10 and days 21–28 after infection. The blood collection time in our study was determined by combining the situation and experiences from literature reports [7,8]. These may lead to a time shift for antibody detection; (3) other reasons such as quantity and quality of the specimens.

In terms of cytokines, our research found that it showed a Th2-dominated cellular immune response based on the increasing of IL-5 expression and decreasing of INF-γ expression after inactivated vaccination. For IL-4, although it showed a statistically significant upward trend with the Kruskal–Wallis H test, the difference was not statistically significant when compared in pairs. This may be caused by the quantity and quality of specimens in our study. In addition, the empirical analysis demonstrated that compared with ELISpot, flow cytometry and ELISA platforms were considered insufficient in IL-4 detection. In our study, we used FCM instead of ELISpot to test cytokines because we had available a proven flow cytometry platform. Current mainstream vaccines generate Th1-dominated cellular immune responses such as AZD1222 (viral vector vaccine, UK), BNT162b1 (mRNA vaccine, USA), and mRNA-1273(mRNA vaccine, USA) [10,11,12]. In the field of inactivated vaccines, currently, only the BBV152 vaccine has claimed that it induced Th1-dominated cellular immune responses. This vaccine adjuvanted with aluminum hydroxide gel (Algel), or TLR7/8 agonist chemisorbed Algel showed Th1-dominated cellular immune response ability [13]. In China, the Sinopharm vaccine used traditional beta-propiolactone-inactivated aluminum hydroxide-adjuvants [8,14]; Sinovas vaccine was also adjuvanted with alum adjuvant which specifically induced Th2-dominated cellular immune response [7]. It is known that aluminum adjuvants enhance the adaptive immune response mediated by Th2 cells and activate the function of B lymphocytes to induce antibody production. This can reasonably explain the Th2-dominated cellular immune response after vaccination in our study. As for the comparison between the two vaccines, we found that the Sinopharm vaccine showed a Th2 cell response type (IFN-γ decreased and IL-5 increased), while the Sinovas vaccine showed a Th2 type cell response (IL-5 increased and IL-8 increased). In the study of the Sinopharm vaccine, notable changes in various cytokines (including T helper 2 cell-related cytokines IL-4, IL-5, and IL-10) were not observed [9]. In addition, we also found that vaccination caused the increase in IL-6 expression, which may be caused by the activation of Th2 lymphocytes. IL-6 supported the growth of B cells and had an antagonistic effect on Treg cells. The Th2-dominated cellular immune response promoted humoral immunity and the production of antibodies. Furthermore, vaccination also caused a certain cellular immune response. Studies have shown that for effective protection against SARS-CoV-2, both antibody-mediated humoral immunity and T cell-mediated cellular immunity are required [15,16].

We found that IL-6 level was directly proportional to antibody level, and INF-γ was inversely proportional to antibody level. These results further verified that the Th2-dominated cellular immune response caused by the inactivated vaccine promoted the secretion of IL-6 to stimulate antibody production. On the other hand, the Th1-dominated cellular immune response was inhibited by suppressing the secretion of INF-γ. In addition, the high levels of IL-8 and IL-12P70 on D14 were related to the increasing of antibodies. IL-12P70 as a key cytokine to Th1-dominated cellular immune response can activate B lymphocytes and stimulate antibody production; meanwhile, it seemed that the vaccination caused a mild Th1 cellular response. Th1 cells produce inflammatory cytokines and participated in cellular immune response, whereas Th2 cells secrete help B cells produce antibodies, a balanced T cell response is critical for safe COVID-19 vaccine development [17]. Therefore, the immune response type induced by the inactivated vaccines in our study was mainly Th2- biased response supplemented with mild Th1-type immunity. Experiences from SARS-CoV and MERS-CoV strongly indicated that Th1-biased response was the key for successful control of coronavirus as well as SARS-CoV-2 [18]. Both Th1 and Th2 type responses were necessary to SARS-CoV-2 infection resistance. The specific mechanism needs to be further explored.

In terms of basal lymphocyte subsets, inactivated vaccines induced certain cellular responses manifested as increases of CD3+, CD8+, and CD56+ cells. However, it was interesting that although the vaccination triggered the production of antibodies, we observed a decrease in CD19+ B lymphocytes. Although the vaccines were inactivated, they still triggered cellular immune responses after vaccination, including increases of T, Ts, and NK cells. Earlier studies on COVID-19 infections confirmed that virus invasion induces innate immunity [19]. In studies of mRNA vaccines, BNT162b1 stimulates strong CD4+ T cell and CD8+ T cell responses and strong antibody responses [20]. A number of clinical trials, including adenovirus-vectored vaccines, inactivated vaccines and mRNA vaccines, confirmed that T cell response was activated. Two adenovirus-vectored vaccines (CanSino Biologics, AstraZeneca) were reported to stimulate CD4+T cell response; mRNA vaccine (Pfizer/BioNTech), mRNA vaccine (Moderna), and inactivated vaccine BBV152 from India simultaneously activate CD4+ and CD8+T cell response after vaccination [11,12,21,22,23]. Literature has confirmed that CD8+T lymphocytes have clear preventive effects on COVID-19 [24]. Other researchers showed that it leads to stronger CD8+T-cell responses in patients with mild COVID-19 disease rather than the severe ones [25]. The role of CD8+ responses is unclear; studies into the cellular immune response to vaccines were necessary [26]. Sinovac vaccine clearly pointed out its limitations in Phase 2 clinical summary: the study design did not include assessment of immune reactions mediated by CD8 cells because inactivated vaccines were not thought to induce CD8 T-cell responses [7]. Our findings seemed to be inconsistent with this speculation, but we could not prove that the CD8 T-cell response was vaccine-specific.

In addition, it showed that CD8+CD71+ and CD56+CD71+ cells were increased, suggesting that the increased T lymphocytes and NK cells in the basic subsets were in the functionally activated state. To our surprise, activation-related cells as CD8+CD38+, CD4+HLA-DR+, and CD8+HLA-DR+ showed a downward trend, which seemed to be the opposite of the CD71-marked activation trend. The mechanism here needs to be further explored. In the B lymphocyte subset, Breg cells increased after vaccination, suggested that B cells initiated the antibody production process. In a comparison of the lymphocyte subsets between the two vaccines, the Sinopharm vaccine showed a higher level on T and B subsets, and the Sinovas vaccine showed a higher level on T and NK subsets.

We found that the ratios of B lymphocytes and DPT cells were inversely proportional to antibody levels. In our subset analysis, we also obtained the result of the decrease in the proportion of B lymphocytes. We believe that the result was due to the differentiation of a large number of B lymphocytes into plasma cells during the continuous production of antibodies. In the analysis of plasma cells, although an increase in the proportion of plasma cells in D42 was observed, this change was not statistically significant, and the speculation needs further studies. However, the increase in Breg cells still suggested that B lymphocytes were in an activated functional state. Participants with a higher proportion of memory B lymphocytes (CD19+CD27+) on D14 were more likely to obtain continuously rising antibody levels, while participants with higher activated Ts cells and naive B lymphocytes (CD19+CD27−) were more likely to have obstacles in maintaining the antibody levels. On D42, participants with a higher proportion of activated Th cells (CD4+CD38+) were more capable of maintaining high antibody levels. Our research demonstrated that although theoretically the inactivated vaccine only stimulates the humoral immunity, it actually triggered different levels of T lymphocyte response and achieved certain cellular immunity.

Of course, this study had certain limitations. The research was an observation based on the immune response after vaccination by detecting various basic indicators and immune indicators. However, references on inactivated vaccine-related lymphocyte subsets are too numbered for us to compare and analyze horizontally; thus, we only presented and discussed the lymphocyte subset changes after vaccination based on the function. We look forward to more subsequent comparative analyses based on these results by other researchers in this field. On the other hand, due to some difficulties of funding and recruitment of participants, the study only included numbered participants, which may not fully reflect the state of the immune response.

Recently, China has carried out large-scale vaccination with the purpose of achieving vaccine-based herd immunity. According to the latest survey of vaccination willingness in the Chinese population, 9.0% of citizens still refused to receive vaccination, and 35.5% reported hesitancy. One large reason was lacking understanding of vaccine information [27]. In the USA, it was reported that individuals’ willingness to receive a vaccine was positively correlated with vaccine efficacy [28]. Therefore, it was important to obtain sufficient vaccine information through relevant research. In this research, for the first time, we fully demonstrated the immune response state brought by inactivated vaccines and compared the differences between two inactivated vaccines. It suggested that the inactivated vaccine not only stimulated the humoral immunity by producing neutralizing antibodies but also induced cellular immunity. The cellular immunity induced by these two vaccines was a Th2-biased response, and it may also lead to weak Th1-type responses.

## Figures and Tables

**Table 1:** Baseline characteristics of participants.

	Sinopharm	Sinovas	Total
Age, years			
20–29	2	1	3
30–39	9	18	27
40–49	1	2	3
Median	33	35	34
Sex			
Male	4	3	7
Female	8	18	26
Total	12 (36.36%)	21 (63.64%)	33

## Data Availability

All data generated or analyzed during this study are included in this.

## Supplementary Materials

The following are available online at https://www.mdpi.com/article/10.3390/vaccines9091028/s1, Table S1: Seroconversion rate compared with literature.
