# Supplementary material for: Immunological Analysis of People in Northeast China after SARS-CoV-2 Inactivated Vaccine Injection
Source: Vaccines (Basel). 2021 Sep 16;9(9):1028. doi: 10.3390/vaccines9091028 (PMC8473348; doi:10.3390/vaccines9091028)
Supplement: Supplementary file 1 [file vaccines-09-01028-s001.zip › vaccines-1339633-supplementary.pdf]

Table S1 Seroconversion rate compared with literatures

	Seroconversion rate in this study		Seroconversion rate in literatures	
	D14	D42	D14	D28
Sinovas	50%	85%	92.4%[7]	94.1-97.4%[7]
Sinopharm	83.3%	58.3%	75%[8]	100%[8]
